# Amputation of the Superior Maxillary, Malar and Palate Bones, for Disease of the Antrum

**Published:** 1846-08

**Authors:** Daniel Brainard

**Affiliations:** Professor of Surgery in the Rush Med. Col.


ARTICLE III.

*Amputation of the Superior Maxillary, Malar and Palate bones, for Disease of the Antrum. Recovery.* By DANIEL BRAINARD, M. D., Professor of Surgery in the Rush Med. Col.

On the 16th of May, 1846, I was requested by my friend, Dr. Philip Maxwell, of this place, to consult with him in the case of Mary Derry, wife of Philip Derry, of Aux Plaines, Ill. She is 40 years of age, of good constitution, and is not subject to any hereditary predisposition to disease.

Present state. Upon the left side of the face, there exists a tumor of the size of an orange, extending from the orbit to the angle of the mouth, and from the nose to the outer part of the cheek. It also projects downward into the mouth, effacing the alveolar process and projecting the cheek, but not encroaching upon the soft palate or extending to the median line of the mouth. Its surface is red and highly vascular, it is painful particularly beneath the orbit, is firm, but slightly and obscurely elastic.

History. Near four years since, Mrs. Derry began to be afflicted with "gatherings in her head," when, after having pain in the left side of the face and head three or four days, there took place a free discharge from the corresponding nostril, and the pain was relieved. This continued to recur from time to time, until the autumn of 1845, when the discharge ceased to recur, the pain, however, continuing and becoming more severe.

In Jan., 1846, she for the first time perceived a swelling of the cheek, and applying to a physician, he removed one or two teeth which were loose, telling the patient, she had an "ulcer tooth." From Jan. to the present time, the tumefaction and pain have increased, so that the patient obtained no rest without the use of anodynes. Since the first occurrence of the pain in her head, her general health remained pretty good until the autumn of 1845, when the menses ceased, and soon after, the pain and want of rest, induced considerable emaciation. All the principal functions of the system with the exception of those just mentioned, are performed with considerable regularity.

Operation. This was performed on the morning of the 23d of May, with the assistance of Drs. Maxwell & Herrick, and in presence of Drs. Kimberly, Boone, Dyer, and several other physicians and medical students, as follows: The patient was placed upon a bed on her back, with the head raised. An incision was then made from the internal angle of the eye to the mouth, dividing the left half of the upper lip at its middle. Another was carried from the upper end of this, in a curve, to the external angle of the eye, and from this point to the eminence of the zygomatic arch. The integuments were dissected up so as to form a broad flap and entirely expose the tumor. This dissection was somewhat difficult, and required great care from the thinning of the skin, which had taken place over the most prominent part of the diseased structure. The lip and ala of the nose were then dissected up, and the incisor tooth next the median line extracted. The next part of the operation consisted in detaching the diseased mass. With a common narrow saw of the amputating case, introduced into the nostril, the alveolar and palatine processes of the superior maxillary bone, and the palate portion of the palate bone, were easily divided, as far back as the soft palate. The nasal process of the maxillary, the connexions of the malar bone to the external angular process of the os frontis, and zygomatic process of the temporal bone, were then divided with the bone scissors, leaving only a posterior bony attachment. To divide this a chisel about one inch wide was placed in the temporal fossa, and with a couple of blows of the hammer it was entirely loosened. It only remained to divide the soft tissues below the orbit and the vail of the palate, at its attachment to the bone, and the whole mass was removed.

Immediately a sponge dipped in cold water was thrust into the wound to arrest the hemorrhage, which was very abundant from a great number of small vessels. In a few minutes this was arrested, with the exception of that from the division of the internal maxillary artery at the bottom of the wound; this required the actual cautery. No ligatures were used. On examining the wound the pterygoid processes were found exposed, and no traces of the diseased tissue remaining. The flap was then brought in place, and retained by about a dozen stitches of interrupted suture. No other dressings were applied, lest by their pressure they should interrupt the circulation in the flap.

The time occupied in removing the tumor, was, from the commencement of the operation, not over from five to ten minutes. It was, however a severe one, and the patient, from the shock and loss of blood, was considerably depressed. Opium was freely given until pain and restlessness were allayed. This was required to be repeated for four days, in order to procure sleep at night. No unfavorable symptoms occurred; the flap adhered throughout by the first intention, and on the fifth day after the operation the menses returned. From this time there was no pain, her appetite and digestion were good, and at the end of a week from the operation, she was able to sit in a chair, walk about the house, and wash the wound herself. Healthy suppuration and granulation took place and on the 15th of June she rode home, about 12 miles, without difficulty, and I saw her there on the 16th, extremely comfortable in every respect.

On laying open the tumor it was found to be composed of a firm fibrous mass nearly as dense as scirrhus in its crude state. There were several small cavities in it, containing healthy pus, but no trace of bony structure. On the outside it was found to be circumscribed on every side by healthy structures, showing that the whole of the diseased mass was removed. A microscopical examination of the tissue showed it to contain "granules and nucleated cells," like those described and figured as characteristic of malignant growths. Under these circumstances, the return of the disease is of course greatly to be feared. The patient has been placed upon such a regimen as to guard as much as possible against this, and we may at least reasonably hope that its return may thus be so far retarded as to give a respite of several years, from so dreadful a malady.

